# Gestational Diabetes Mellitus—Innovative Approach to Prediction, Diagnosis, Management, and Prevention of Future NCD—Mother and Offspring

**DOI:** 10.3389/fendo.2020.614533

**Published:** 2020-12-03

**Authors:** H. David McIntyre, Anil Kapur, Hema Divakar, Moshe Hod

**Affiliations:** ^1^Mater Research, The University of Queensland, South Brisbane, QLD, Australia; ^2^World Diabetes Foundation, Bagsvaerd, Denmark; ^3^Divakars Specialty Hospital, Bengaluru, India; ^4^Mor Women’s Health Care Center, Tel Aviv, Israel

**Keywords:** gestational diabetes, prediction, diagnosis, prevention, management, pregnancy, non-communicable disease

## Abstract

Gestational diabetes mellitus (GDM) is the commonest medical complication of pregnancy. The association of GDM with immediate pregnancy complications including excess fetal growth and adiposity with subsequent risk of birth trauma and with hypertensive disorders of pregnancy is well recognized. However, the associations with wide ranges of longer-term health outcomes for mother and baby, including the lifetime risks of obesity, pre-diabetes, and diabetes and cardiovascular disease have received less attention and few health systems address these important issues in a systematic way. This article reviews historical and recent data regarding prediction of GDM using demographic, clinical, and biochemical parameters. We evaluate current and potential future diagnostic approaches designed to most effectively identify GDM and extend this analysis into a critical evaluation of lifestyle and nutritional/pharmacologic interventions designed to prevent the development of GDM. The general approach to management of GDM during pregnancy is then discussed and the major final focus of the article revolves around the importance of a GDM diagnosis as a future marker of the risk of non-communicable disease (NCD), in particular pre-diabetes, diabetes, and cardiovascular disease, both in mother and offspring.

## Background

On a worldwide basis, both Hyperglycemia in Pregnancy (HIP) and obesity among women of childbearing age are increasing to epidemic proportions (1, 2). For our current report, we are following the diagnostic framework for HIP as recommended by the International Federation of Gynecology and Obstetrics (FIGO) (3) which considers any degree of glucose elevation in pregnancy as part of the umbrella definition of HIP. This broad group is then further divided into those women with either known pre-pregnancy diabetes or markedly elevated glucose levels which would qualify for a diagnosis of diabetes outside pregnancy. This condition is termed “Diabetes in Pregnancy” (DIP). The far larger group of women with elevated glucose levels below these thresholds is classified as “Gestational Diabetes Mellitus” (GDM). Many reports have attempted to dissect the relative importance of HIP and overweight/obesity relating both to pregnancy complications and longer term health of both GDM mothers and their children. Clear determination of causality is challenging, GDM and overweight/obesity frequently affect the same people, and causality is bidirectional—obesity lies on the causal pathway toward hyperglycemia and HIP is causally related to obesity in the offspring. Also, hyperglycemia (pre-diabetes or diabetes) may well exist before pregnancy as demonstrated by large surveys such as NHANES (4, 5). However, it is generally asymptomatic at this point and may only be detected when (and if) systematic testing occurs in the pregnancy context.

The Hyperglycemia and Adverse Pregnancy Outcome (HAPO) study (6, 7), demonstrated that both maternal BMI and hyperglycemia share similar associations with complications of pregnancy. Both were associated with increased rates of excess fetal growth, primary cesarean birth, clinical neonatal hypoglycemia and fetal adiposity, neonatal hyperinsulinemia, and hypertensive disorder of pregnancy. The association of hyperglycemia with adverse outcomes is generally linear, while that of BMI follows a quadratic pattern with decreasing increments in the highest BMI categories (8).

HAPO also reported BMI and GDM considered together with pregnancy complications (6). In HAPO, obesity was present in 13.7% and GDM by IADPSG criteria (9) in 16.1% of those who remained blinded. Obesity was present in 25% of GDM women but prevalence varied greatly between centers. Compared to women with neither factor, adverse outcomes were increased on both groups. Pre-eclampsia was more frequent in the “obesity/non GDM group”, while excess fetal growth and fetal hyperinsulinemia were slightly more common in the “GDM/no obesity group” than in the “obesity/non GDM group”. The combination of both factors was accompanied by an additive increase in pregnancy complications.

Thus, both maternal BMI and glycemia contribute independently and additively to suboptimal pregnancy outcomes. While not ignoring obesity as a major health problem, our current review will focus primarily on the prediction and diagnosis of GDM and outline optimal management both during and following pregnancy.

## Prediction of GDM

As noted above, many women currently considered as “GDM” may actually have undiagnosed hyperglycemia before pregnancy. In high prevalence countries, ideally screening for hyperglycemia would happen as part of well-organized and well- resourced preconception care. However, this approach has limitations as only around 40% of pregnancies worldwide are “intended” (10). Lack of preconception testing means that we cannot strictly assert that testing in early pregnancy is “predicting” GDM. However, early testing does represent an opportunity to recognize some women as having likely pre-existing abnormalities of glucose metabolism. In India, for example, it has been reported that over 70% of GDM women can be identified at their first antenatal visit (11).

Further, it may be possible, by using clinical characteristics and biochemical tests, to identify a separate group of pregnant women whose glucose levels are in the normal range in early gestation, but who carry a high risk of progression to “standard GDM”, which is generally diagnosed at around 24 to 28 weeks’ gestation. In pragmatic terms, it appears reasonable to consider women with pre-pregnancy hyperglycemia, those with early GDM and those at high risk of GDM as target groups for early intervention.

GDM is frequently an antecedent of later Type 2 diabetes and a marker for (premature) cardiovascular (CV) disease in women. In addition to their *sine qua non* of hyperglycemia, GDM and Type 2 diabetes share a range of underlying processes including insulin resistance, chronic metabolic inflammation, changes in adipocytokines, and alterations in many areas of metabolism (12, 13).

The simplest models for prediction of GDM involve the use of single or multiple clinical characteristics to stratify GDM risk. The performance of these models has recently been evaluated by van Hoorn et al. (14), who concluded that models which included both multiple clinical characteristics and early pregnancy glucose measurements performed best in prediction. Recently, machine learning or artificial intelligence methods using demographic variables and previous laboratory results have been applied to improve predictive power (15).

GDM is also associated with abnormalities of placentation and early pregnancy markers commonly used in aneuploidy prediction such as pregnancy associated plasma protein A (PAPP-A) and free β HCG have also been incorporated into predictive models (16). Sweeting et al., using stored serum samples from a first trimester screening program in Sydney, have reported that use of multiple biochemical markers in combination with clinical features, is able to predict GDM with high accuracy [area under receiver operator curve (AUROC) 0.91–0.93]. However, these findings have yet to be validated in independent cohorts.

Proteomic screening in early pregnancy has revealed multiple potential protein markers, including a cluster associated with insulin secretion, binding, resistance, and signaling for later GDM (17, 18). Ravnsborg et al. have reported that vitronectin, which is also associated with metabolic syndrome outside pregnancy, significantly augments the predictive power of maternal risk factors (17) and may prove to be a valuable predictor in clinical use. However, proteomic methods are too complex and expensive for routine use and must progress to automated, low cost laboratory tests before they are widely applicable.

Recently, the role of extracellular vesicles (ECVs) as GDM markers has been explored (19, 20). These circulating particles, derived in pregnancy primarily from placenta and adipose tissue, “package” multiple potential protein and RNA molecules and transport them to specific sites. James-Allan et al. (21) have demonstrated that specific small ECVs are associated with GDM and that infusion of human ECVs from GDM women produces both insulin resistance and reduced insulin secretion in rodents, reminiscent of the pathophysiology of GDM.

Micro RNAs are a major component in ECVs and are associated with glucose metabolism. An exploratory case-control study by Yoffe et al. suggested that micro RNA-223 and micro RNA 23a in first trimester blood samples were strongly predictive of later GDM (AUROC 0.91) (22). Another recent cohort study has confirmed this finding for micro RNA-233 (23). These results are promising and the overall associations between non coding RNAs and GDM have recently been reviewed in detail (24). However, as is the case for other biomarkers, these positive findings from small studies need to be validated in independent cohorts. The required assays will also need to be modified to allow low cost, high throughput use in routine diagnostic laboratories.

In summary, cohort studies have revealed multiple potential early pregnancy predictors of later GDM (13). These range from single or multiple clinical or demographic measures to including early pregnancy glycemic measurement and extending to measurement of complex network of molecular biomarkers. To be valuable for routine clinical practice, molecular biomarkers need both to perform better than clinical risk factors and simple glucose measurements in predicting GDM and pregnancy outcomes and to demonstrate cost-effectiveness. In practical terms, they should also be suited to non-fasting testing at the same time as other routine early pregnancy health screening tests. While many biomarkers have a strong association with later GDM, none have yet been sufficiently developed as automated and low-cost assays to allow their routine clinical use. However, they offer valuable insights into the pathophysiology of GDM and may, in time, be ready for the clinic.

## Diagnosis of GDM

GDM is generally diagnosed with an oral glucose tolerance test (OGTT) administered at 24–28 weeks’ gestation. This timing has generally been preferred for routine GDM diagnosis as most of the physiologic insulin resistance of pregnancy will be well established. However, with globally increasing levels of obesity, rising maternal age and other environmental risk factors this assumption may no longer be valid as evidenced by high GDM detection rates in early pregnancy witnessed in recent studies from different parts of the world. The rising prevalence of undiagnosed dysglycaemia (diabetes and pre-diabetes) in reproductive age women enhances the need to rule out pre-existing undiagnosed diabetes at the earliest possible moment, thus bringing into question the old norm of testing between 24 and 28 weeks. The exact process and criteria for OGTT diagnosis of GDM vary widely across the world. The International Association of Diabetes in Pregnancy Study Groups (IADPSG) (9), World Health Organization (WHO) (2, 25) and FIGO (3) have all endorsed “one step” OGTT testing, using thresholds ≥ 5.1 mmol/L fasting; 10.0 mmol/L at 1 h and 8.5 mmol/L at 2 h following a 75 gram glucose load for diagnosis of GDM. However, the FIGO pragmatic recommendations, recognizing differing health care contexts, allow for alternative diagnostic approaches for China, India, South America, and the United Kingdom (3). There is a major variance in diagnostic approach in the USA (26) and Canada (27) which generally prefer two step testing using a non-fasting, 1 h “glucose challenge” test (GCT), followed by OGTT (100 gram or 75 gram) if the GCT result falls above predefined thresholds. IADPSG, WHO, and FIGO have all also endorsed the need for early testing as well as testing in the traditional 24 to 28 week window.

A further major variation in worldwide testing protocols for GDM is the ongoing debate regarding whether testing should be universal (for all pregnant women) or targeted only toward women with identified risk factors which are associated with a higher risk of a positive test. FIGO (3), the IADPSG (9) and the American College of Obstetricians and Gynecologists (ACOG) (26) all recommend universal testing. The HAPO study (7) clearly demonstrated that OGTT glucose values are independently associated with adverse pregnancy outcomes, even after correction for multiple additional maternal characteristics including BMI. GDM is almost uniformly asymptomatic, so a testing strategy based on symptoms is clearly untenable. A cost utility analysis for the United Kingdom (UK) concluded that if the population frequency of GDM is >4.2%, then universal OGTT is the most cost effective strategy (28). No current credible estimates of GDM frequency fall below this threshold. Nonetheless, some authorities, notably the National Institute for Clinical Excellence (NICE) in the UK, still promote risk factor based screening (29). The most recent Cochrane review (30) is inconclusive, but a recent systematic review of economic evaluations of GDM screening again concluded that universal screening was the most effective approach (31). Compliance with officially endorsed risk factor based screening protocols appears to be poor in countries as diverse as Sweden (32), where only 31% of women received the screening test deemed appropriate for their documented risk factor profile, the UK (61% appropriate screening according to risk factors) (33) and South Africa (34), where Adam et al. reported that, although use of risk factors would reduce OGTTs by 46%, this protocol would miss 41% of GDM diagnoses (34). A further study from Italy reported that 23% of GDM cases would be missed by risk factor based screening (34). By contrast, a report from Sri Lanka, a country with a high background frequency of diabetes, noted that 80% of women would still require screening if risk factors were used to determine the need for OGTT, but only 13% of GDM cases would be missed (35). Thus, the effects of implementing risk factor–based screening differ between various populations. However, given that most countries have an increasing prevalence of both GDM and associated risk factors and that correct implementation of risk factor based screening is poor, we favor uniform biochemical testing.

Clearly, the glucose tolerance test is inconvenient, resource intensive and also quite poorly reproducible (36) and an alternative cheap, reproducible, non-fasting test would be preferable. Self-administered home OGTTs offer increased convenience and appear to perform as well as laboratory testing (37). For fasting glucose testing, specific meters with tight laboratory based quality control may also provide acceptable accuracy (38) and this approach has been endorsed by FIGO for use in low resource settings (3).

Glycosylated haemoglobin (HbA1c) is an obvious alternative and is widely used for diagnosis of diabetes outside pregnancy. However, it performs poorly both in prediction of OGTT diagnosed GDM and in prediction of pregnancy outcomes (39) and appears to be of limited value except in early pregnancy detection of undiagnosed hyperglycemia (40).

A variety of other markers of overall glycemia with shorter half-lives (thus more reflective of short term glycemia changes) including fructosamine (FA), glycated albumin (GA) and 1.5 anhydroglucitrol (1.5 AHG) have been evaluated (41). None of these markers are optimally suited to use in pregnancy. FA is easily measured but is affected by the dilutional anaemia of pregnancy. FA and GA vary in the presence of albuminuria (as in pre-eclampsia) and 1.5 AHG is inaccurate in pregnancy due to reduced renal threshold for glycosuria (41). More recently, the glycated complement fraction GCD59 (42) has been shown in retrospective studies to be associated with early GDM and with large for gestational age (LGA) infants (43) in an obese pregnant population. Larger prospective evaluations are now underway (44). GCD59 is currently the most promising, non-fasting marker of overall glycemia in the context of pregnancy.

## Prevention of GDM—Before and During Pregnancy

Ideally, GDM rates in the population could be reduced by both individual and societal measures designed to promote healthy lifestyle changes including optimal dietary intake and increased physical activity in the general population with a focus on health and fitness of women of reproductive age. Maternal age at conception is an important marker of pregnancy complications including GDM (45), but is strongly influenced both by individual choices and societal factors and unlikely to be a useful target for preventative measures. Maternal overweight and obesity are also very important risks that need to be addressed pre-pregnancy through lifestyle measures (45). For marked obesity, bariatric surgery is the best proven means of reducing body weight. Although a full consideration of its effects on pregnancy are beyond the scope of this article, a recent review (46) showed benefits reduced GDM prevalence, lower rates of LGA infants and reduced prevalence of pregnancy hypertension. However, there was an increase in impaired fetal growth reflected by higher rates of SGA infants. Preterm deliveries were also more frequent. Surgical treatments which cause malabsorption are followed by greater weight reduction and demonstrate lower rates of LGA, but conversely also more SGA, suggesting that clinical decisions must balance potential risks and benefits.

The most recent overview of multiple GDM prevention strategies from the Cochrane group (47) reports that no single lifestyle or medication-based intervention is of proven benefit and that the overall evidence base is weak, but we shall consider some widely discussed potential options in more detail.

## Lifestyle Interventions

Despite the negative Cochrane findings outlined above, which specifically concluded that diet alone or exercise alone had no effect in preventing GDM and that combined diet and exercise had only a “possible” (non-significant) effect in preventing GDM, one systematic review by Song et al. (48) has suggested that lifestyle intervention before the 15^th^ gestational week may reduce GDM by 20% [RR 0.80 (95% 9I 0.66–0.97)]. This provides glimmer of hope for lifestyle based interventions, but the weight of evidence is negative for any lifestyle intervention after the first trimester.

## Metformin

Metformin is commonly used in early pregnancy for patients with polycystic ovarian syndrome. It is also used in the second and third trimester as drug treatment for GDM if lifestyle modifications fail to achieve glycemic goals. A recent review has summarized many aspects of metformin use in pregnancy (49). However, current evidence does not support metformin as a preventative option for GDM. The (EMPOWaR) study randomised 449 obese women with normal baseline glucose tolerance to the addition of metformin of up to 2,500 g per day vs. placebo between 12- and 16-week gestation and continued until delivery of the infant (50). EMPOWaR noted no difference GDM prevalence, maternal weight gain or maternal lipid metabolism. Birth weight and birthweight SD (Z) score were comparable between the groups. Metformin was associated with more reported diarrhoea (42% vs 19%, P < 0.0001).

Syngelaki et al. randomized women with BMI > 35 kg/m^2^ to metformin 3 g per day or placebo from 12- to 18-week gestation until delivery (51). A total of 202 women on metformin and 198 on placebo completed the study. There was no difference in fetal growth. Maternal GWG was reduced by 1.7 kg (P < 0.001) with metformin. GDM rates were similar between groups and other pregnancy outcomes were also similar.

The GRoW RCT of metformin for overweight and obese women from early pregnancy, conducted by Dodds et al., has also reported negative results, with no reduction in GDM or other pregnancy complications (52).

## Myoinositol

Myoinositol enhances insulin sensitivity outside pregnancy. In GDM, a trial of supplementation of 69 women randomised to myoinositol 4 g/day with folic acid 400mcg daily compared to folic acid alone showed reduced insulin resistance in the myoinositol group (53). Another study compared myoinositol 2 g with 200 mcg folic acid daily vs. 200 mcg folic acid daily alone in 220 obese women starting at 12- to 13-week gestation and continued throughout pregnancy (54). GDM prevalence was reportedly reduced from 33.6% to 14% (P = 0.001), in the myoinositol group.

However, another RCT which recruited women with family history of diabetes and compared women randomised to 1,100 mg myoinositol, 27.6 mg D-chiro-inositol plus 400 mcg folic acid, or to 400 mcg folic acid only, from early pregnancy until 24- to 28-week gestation demonstrated no reduction in GDM frequency (55).

Resveratrol was compared to inositol in another RCT involving overweight pregnant women. The three arms of the study included resveratrol plus inositol, inositol alone and placebo (56). Resveratrol was associated with reduced lipid and glucose measures. The NiPPeR study plans to compare twice-daily intake of a control nutritional drink, enriched with standard micronutrients, to a twice-daily nutritional drink enriched with additional micronutrients, myoinositol, and probiotics. Results are not yet available (57).

## Probiotics

Probiotics were found reduce GDM in normal weight women in a study from Finland (58), a result which stimulated great interest in their use as a low risk therapeutic agent in GDM prevention. However, results in women at higher risk due to pre-pregnancy obesity have been less promising. The Probiotics in Pregnancy Study randomised 175 obese women to probiotic vs. placebo capsule for a four week period from 24 until 28 weeks gestation, with the primary outcomes being change in fasting glucose. Treatment groups differed in baseline BMI. After adjustment for BMI, there were no discernible clinical benefits in the major outcome of fasting glucose. Infant birthweight and other metabolic parameters did not improve (59). More recently, the SPRING study (60), HUMBA study (61) and studies from Finland (62) and Denmark (63) have reported negative results for probiotic supplementation, so the weight of current evidence is certainly negative.

## Fish Oil

Dietary fatty acid has also been suggested as a therapy which might reduce GDM and also improve the rates of preterm delivery. The DHA to optimize mother infant outcome (DOMInO) RCT included 2399 women who were randomized before 21-week gestation to either (1) DHA enriched fish oil 800 mg/day or (2) vegetable oil capsules without DHA until the time of birth (64). GDM and preeclampsia were not reduced and there were no differences in neonatal size or adiposity. Subsequent assessment of the offspring at Age 7 years showed no differences in anthropometry (65).

## Vitamin D

Low serum 25 hydroxy vitamin D levels are clearly a risk factor for development of GDM (66), but the results of therapeutic trials have been mixed. The most recent Cochrane review (67), including primarily studies from the Middle East, reported that supplementation with Vitamin D alone “probably” reduces the population frequency of GDM [RR 0.51 (95% CI 0.27–0.97)] and pre-eclampsia [RR 0.48 (95% CI 0.30–0.79)]. However, no benefit was noted for Vitamin D + calcium or Vitamin D + calcium + other minerals. The overall quality of available evidence has been considered “low” by the Cochrane Group in their overview of GDM prevention studies (47). Noting the overall divergence in populations studied, baseline Vitamin D levels and therapeutic Vitamin D doses used in the studies included in/excluded from various reviews, we consider the following conclusion from Corcoy et al. (68) as a balanced summary: “High dose vitamin D supplementation during pregnancy halves the rate of GDM in pregnant women with baseline Vitamin D < 50 nmol/L”. Toxicity from Vitamin D supplementation up to 5,000 IU/day appears low (69, 70). Therefore, in practical terms, Vitamin D therefore appears to be a reasonable option in populations with low baseline levels. Hopefully, future studies will clarify its true therapeutic role.

## Management of GDM—During Pregnancy and Post-Partum

Treatment of GDM during pregnancy, centers on dietary modulation, promotion of healthy physical activity and pharmacologic management, primarily with insulin as well as oral hypoglycemic agents (OHA), if glycemic control cannot be achieved with lifestyle measures alone. The details of the therapeutic approach, in particular regarding various dietary approaches and the potential use of OHAs such as metformin and glyburide (glibenclamide) differ widely between and within countries and detailed discussion of these ongoing issues of contention is beyond the scope of our current review, but has recently been covered in detail (71).

Two landmark prospective RCTs have confirmed that detection of and medical therapy for GDM carries benefits for both mother and baby in terms of immediate pregnancy outcomes (72, 73). Women in the intervention arm of the Australian (Crowther) study showed lower rates of fetal macrosomia, reduced frequency of LGA and reduced preeclampsia (72). In the US (Landon) study, women who received treatment for GDM demonstrated lower GWG and lower rates of pre-eclampsia. Reduced frequency of LGA and macrosomia were noted in infants of treated mothers.

## Prevention of Future NCD—Mother and Offspring

Apart from its short term immediate associations with adverse perinatal outcomes, antecedent GDM is the strongest available historical marker for future Type 2 diabetes. GDM women carry an almost tenfold increased risk of progressing to later diagnosis of type 2 diabetes [RR 9.51 (95% CI 7.14–12.67)] (74). Women with GDM are also at heightened risk of CV disease (75). This increased risk exists even in those women who do not progress to overt Type 2 diabetes themselves. In the overall population of women with Type 2 diabetes, those with antecedent GDM also appear to carry higher risks than those without such a history (76). Compared with those who did not have GDM, women with GDM had a twofold higher risk of future CV events [RR 1.98 (95% CI 1.57–2.50)].

Meta-regression analysis demonstrates that this relationship is independent of Type 2 diabetes incidence across various studies (P = 0.34). Even when considering only those women who did progress to overt Type 2 diabetes, GDM was associated with an elevated chance of suffering future CV events [RR 1.56 (95% CI 1.04–2.32)]. Much of this risk occurs early in the life course post pregnancy, with a 2.3-fold increased noted for CV events in the first decade post-partum [RR 2.31 (95% CI 1.57–3.39)] (77).

The therapeutic opportunity for delaying/preventing type 2 diabetes through post-partum lifestyle modifications and possible use of pharmacotherapy in women identified as being “at risk” due to prior GDM (78) has been conclusively shown. A German study reported that breastfeeding (BF) was associated with a > 40% reduction in diabetes also appeared to delay the onset of T2DM by a further 10 years. These effects were not dependent on known high risk maternal characteristics such as baseline obesity or a need for insulin treatment during the index pregnancy. The greatest reduction in later diabetes risk was seen in women who breast fed for >3 months (79). A systematic review and meta-analysis of the association of BF with the post-partum risk of progression from GDM to overt type 2 diabetes reported reduced risks for both pre-diabetes and T2DM. Pre-DM was substantially reduced (OR = 0.66; 95% CI, 0.51–0.86 and T2DM was also less frequent (OR = 0.79; 95% CI, 0.68–0.92). Positive effects were noted among women with longer duration of any BF following a GDM pregnancy. These were even more evident with longer follow-up. Compared with women with shorter duration of BF, longer BF duration was associated with improvement in glucometabolic parameters, including lower fasting glucose and enhanced insulin sensitivity, lower BMI at follow up improved lipid metabolism as demonstrated by lower triglyceride levels. Thus, BF is potentially of great value as a low cost preventative measure in preventing both T2DM and related metabolic derangements in women with a history of GDM, in addition to its other well recognized benefits (80). Evidence for prevention of CV disease in women with history of GDM through post-partum interventions is currently lacking; but logically breast feeding and improved post-partum lifestyle would be predicted to reduce the risk significantly.

Excessive increase in maternal weight from one pregnancy to the next is often associated with a failure to return to pre-pregnancy weight. This in turn is clearly related to a higher frequency of adverse outcomes in subsequent pregnancies. Documented risks include higher rates of stillbirth (81). Further this ongoing weight gain adds to the “vicious cycle” of weight gain and heightened risks of development of T2DM and CV disease. However, the precise contribution of interpregnancy weight gain to the overall CV risk profile been quantified in clinical studies.

Offspring developing in a hyperglycemic environment *in utero*, carry higher risks of obesity developing early in the life course, progression to early impaired glucose tolerance, later development of T2DM and long term risks of development of overt CV disease. These risks are independent of maternal obesity and broadly fall into the conceptual framework of “developmental origins of health and disease” (DoHaD) (82–85). German researchers comparing GDM and non GDM offspring described higher risks for development of childhood disorders, even after adjustment for maternal BMI. They reported adjusted OR of 1.81 (95% CI, 1.23–2.65) for childhood overweight and 2.80 (95% CI, 1.58–4.99) for obesity (83). Maternal GDM also increased the risk of childhood abdominal adiposity (OR, 1.64; 95% CI, 1.16–2.33). Israeli researchers have reported an association between mild GDM (diet treated) and offspring CV morbidity (relative risk, 1.6; 95% CI, 1.2–2.2) (86).

Other researchers have suggested a link between GDM and neuro psychiatric disorders in offspring (87).

The efficacy of current standard GDM interventions during pregnancy in attenuating the risks of obesity and impaired glucose metabolism in children of GDM mothers is much less clear. Existing report suffer from methodologic difficulties related to *post hoc* design and incomplete cohort follow-up. Specifically designed prospective follow up studies would be of great value, but these are clearly difficult to design, difficult to fund and difficult to conduct. Follow up from the landmark GDM RCTs which demonstrated benefits of GDM treatment during pregnancy have been conducted. Gillman et al. (88) reported follow up from the Crowther study (72), but this report was comprised of limited follow up through school based databases rather than direct clinical contact. No benefit was shown for the offspring after maternal treatment of GDM. Landon et al. (89) reported follow up of their North American study (73) and despite detailed clinic visits, they again descried no clear offspring benefits from maternal GDM therapy. Landon et al. study did describe some minor improvement in glucometabolic status for girls whose mothers were treated for GDM. This contrasted with their previous findings related to immediate pregnancy outcomes, which had suggested that males were more likely to benefit from GDM treatment (90). The reason for this disparity remains unclear.

Gunderson et al., describing a cohort based in the USA, have recently reported that breast feeding may reduce some of the offspring risks associated with maternal GDM. They note that offspring weight for length Z score was reduced by 0.36–0.45 standard deviation units at 12 months of Age in those babies of GDM mothers who were intensively breast fed (91). At this stage, data regarding potential longer term effects is not available.

## Post-Partum Glucose Testing

All women with HIP (GDM and overt DIP), should have their glycemic status reassessed with a 75 g OGTT at 6–12 weeks after delivery (3). However, compliance with this recommendation is poor in most health care systems (92) and alternative strategies based on testing in the early post-partum period are supported to some extent by physiologic (93) research demonstrating that changes in maternal insulin sensitivity occur rapidly following delivery. Early testing appears adequate to exclude ongoing Type 2 diabetes at this time point (94), but appears insufficient to exclude impaired fasting glucose (IFG) or impaired glucose tolerance (IGT) (94). Diagnosis at 6–12 weeks should conform to the local non-pregnant criteria for diabetes, IFG, and IGT. Those women whose test results fall into the normal range at this point should undergo ongoing surveillance for diabetes, while those with abnormal results should receive structured interventions for pre-diabetes or diabetes depending on their findings (95).

Irrespective early post-partum glucose results, all women with GDM should be considered to carry higher risks of future diabetes and CV disease. They should be advised to breast feed as long as possible, institute healthy eating patterns and habitual physical activity and seek to achieve a normal body weight. Ideally, they should receive support through consultation and ongoing follow-up with health care professionals knowledgeable about diabetes prevention. It must be recognized that this post-partum care is often the best (and only) opportunity to attempt to improve overall maternal health before the next pregnancy and represents an important opportunity to influence maternal and infant health over the life course.

The biggest stumbling block in the care of GDM mother and her offspring lies in post-partum follow up and inability to set up programs that can help intergenerational prevention of NCDs. There are many barriers in achieving these objectives (96). Following delivery, women with GDM (usually) no longer have glucose levels in the pre-diabetic or diabetic range. Further, they are no longer pregnancy and thus the maternal health care system rarely provides for ongoing care beyond 6- to 12-week post-partum. Thus, they are less likely to attend for regular follow up of their own health issues. Furthermore, the question of responsibility for ongoing care is often left “open”, without a clear plan for ongoing follow-up (97).

However, baby health care is often a major priority at this stage and women are generally very diligent in ensuring that their baby receives routine health checks, growth and developmental assessments and scheduled vaccinations. These often occur in a well-organized fashion for at least five years after birth (98). Therefore, obstetricians, family physicians, internists, and pediatricians must develop systems to link post-partum follow up women with GDM with the recommended routine care of their child as this appears to give the best chance of high quality care and health care engagement for the mother-baby dyad (3).

All offspring of mothers with HIP are at a heightened risk of glucometabolic and CV disease. However, the female offspring carry the additional risk of developing HIP themselves when they reach reproductive age and thus compounding the intergenerational “vicious cycle” of NCD transmission. Pregnant women with a maternal GDM history themselves are at higher risk of GDM compared to those with such a history from the paternal side (99). It is therefore important to test these women for hyperglycemia before conception or as early as possible during pregnancy and repeat testing in each trimester.

## Summary

In summary, GDM represents a major challenge both in the short and long term. In the immediate pregnancy context, GDM detection and treatment are clearly valuable in improving outcomes. The associations with long term health of mothers and babies are also clear, but the optimal approach to treatment remains to be demonstrated. This is a global problem! Prevention and intervention, both during and following pregnancy are urgently needed to reduce the NCD burden of GDM as manifest for affected women and for their offspring. Despite expanding knowledge in this area, practical implementation of proven strategies remains limited. There is great potential for reduction in NCD burden from widespread implementation of relatively simple strategies, to stem the tide of the “slow motion disaster” of obesity and diabetes as identified by Doctor Margaret Chan, Director of the World Health Organization, in 2017 (100).

## Author Contributions

HM wrote the first draft of the manuscript and is guarantor of the work. AK, HD, and MH all reviewed and edited the manuscript and approved the final draft. All authors contributed to the article and approved the submitted version.

## Conflict of Interest

The authors declare that the research was conducted in the absence of any commercial or financial relationships that could be construed as a potential conflict of interest.
